# Impact of nutrition counseling on dietary improvements, glycemic control, and neonatal outcomes in pregnant women with pregestational diabetes mellitus: an electronic medical charts analysis study in a tertiary medical center

**DOI:** 10.1186/s12884-025-08403-4

**Published:** 2025-11-11

**Authors:** Ya-Ling Wang, Chiao-Ming Chen, Chia-Chun Chung, Wei-Chen Chen, Jui-Hua Liao, Wen-Ching Yang, Hsiu-Wei Su, Xin-Yu Chiang, Sing-Chung Li

**Affiliations:** 1 Department of Food and Nutrition, Taichung Veterans General Hospital, Taichung, 407219 Taiwan; 2 Department of Nutrition (Master Program), Hungkuang University, Taichung, 433304 Taiwan; 3 Department of Food Science, Nutrition, and Nutraceutical Biotechnology, Shih Chien University, Taipei city, 10462 Taiwan; 4 Department of Obstetrics, Gynecology, and Women’s Health, Taichung Veterans General Hospital, Taichung, 407219 Taiwan; 5 Department of Obstetrics and Gynecology, Taichung Tzu Chi Hospital, Taichung, 427003 Taiwan; 6 Department of Public Health, Chung Shan Medical University, Taichung, 402306 Taiwan; 7 School of Nutrition and Health Sciences, College of Nutrition, Taipei Medical University, Taipei, 11031 Taiwan

**Keywords:** Nutrition counseling (NC), Pregestational diabetes mellitus (PDM), Pregnancy, Glycemic control

## Abstract

**Background:**

Pregnant women with pregestational diabetes mellitus (PDM) are at higher risk of adverse maternal and neonatal outcomes, including preterm birth and macrosomia. Nutrition counseling plays a critical role in improving glycemic control; however, its effectiveness in pregnant women with PDM in Taiwan has not been fully evaluated. This study aims to evaluate the effects of nutrition counseling on dietary intake, glycemic control, gestational weight gain (GWG), and neonatal outcomes in pregnant women with PDM.

**Methods:**

We conducted a retrospective review of electronic medical records from 2012 to 2022 at a tertiary medical center in Taiwan. A total of 106 pregnant women with type 1 or type 2 diabetes were included, of whom 48 received nutrition counseling (NC group) and 58 did not (Non-NC group). Dietary records, blood glucose, and pregnancy outcomes were analyzed.

**Results:**

There were no significant differences between the groups with and without nutrition counseling in terms of gestational weight gain, preterm birth rate, or the incidence of macrosomia. Nutrition counseling significantly increased the intake of fruits and whole grains and improved dietary fiber consumption (*p* < 0.001). After the intervention, the proportion of women consuming < 175 g of carbohydrates decreased from 55.3% to 34.2%. Among 24 participants with pre- and post-intervention blood glucose data, fasting glucose and HbA1c levels decreased significantly (*p* < 0.001 and *p* = 0.001, respectively). Greater improvements were observed in those receiving multiple counseling sessions.

**Conclusions:**

Nutrition counseling enhanced fiber intake by increasing the consumption of fruits, vegetables, and whole grains. Repeated counseling sessions significantly improved glycemic control, although no significant changes were observed in pregnancy outcomes. These findings underscore the clinical value of integrating structured nutrition counseling into routine prenatal care and highlight the need for larger prospective studies to confirm its impact on pregnancy outcomes.

## Introduction

The prevalence of diabetes has been increasing annually, with a noticeable trend toward younger age groups. Women’s increased access to education and participation in the workforce may contribute to delayed childbearing. In the past, pregnancies among women with diabetes were primarily associated with type 1 diabetes. However, in recent years, there has been a rising trend in pregnancies among women with type 2 diabetes. During pregnancy, the secretion of hormones such as cortisol, progesterone, estrogen, prolactin, and human placental lactogen increases, leading to reduced insulin sensitivity. As a result, mothers who had diabetes before pregnancy may find it even more difficult to control their blood sugar levels [1]. Studies have shown that women with pregestational diabetes mellitus, including type 2 diabetes (T2DM) and type 1 diabetes (T1DM), have a higher risk of adverse pregnancy outcomes compared to the general pregnant population. These outcomes include increased rates of pre-eclampsia, preterm delivery, macrosomia, stillbirth, and congenital malformations [2, 3]. Therefore, they require planned preconception care, including optimization of blood glucose levels, weight and dietary management, as well as postpartum support [4]. The American Diabetes Association (ADA) provides comprehensive recommendations for the management of diabetes in women, particularly during pregnancy. These guidelines emphasize the importance of maintaining optimal blood glucose control to reduce the risk of maternal and fetal complications. When necessary, insulin or other glucose-lowering medications may be used to achieve appropriate glycemic targets. Nutritional management is also highlighted, with a focus on individualized meal planning to ensure sufficient nutrient intake while maintaining stable blood glucose levels. In addition, appropriate weight gain based on individual pre-pregnancy weight status is recommended to support healthy pregnancy outcomes. Regular monitoring and follow-up of both fetal development and maternal health are essential components of effective diabetes management during pregnancy [5].

Nutritional counseling helps provide pregnant women with diabetes with appropriate and individualized dietary recommendations. The goal is to maintain blood glucose levels within the normal range while avoiding hypoglycemia or ketosis that may result from excessive carbohydrate restriction. Additionally, the type of carbohydrate consumed can significantly affect blood glucose concentrations in individuals with diabetes [6]. A recent study of 615 pregnant women with type 1 diabetes (T1DM) showed that those who followed a fresh food–based dietary pattern—including vegetables, fruits, whole grains, lean meats, and low-fat dairy products—had significantly lower HbA1c levels and a markedly reduced risk of pre-eclampsia, preterm birth, and decreased congenital anomalies [7, 8]. Other studies have shown that the intake of high-quality carbohydrates (such as whole grains, legumes, and low-GI foods) and a high-fiber diet can help improve blood glucose control in women with gestational diabetes mellitus, reduce excessive weight gain, and lower the risk of preterm birth and macrosomia [9]. Yang et al. highlighted that even among healthy pregnant women without diabetes, pre-pregnancy body mass index (BMI) and gestational fasting blood glucose (FBG) levels are independently and jointly associated with fetal overgrowth. Their findings suggest that early monitoring and management of maternal metabolic indicators are crucial for promoting healthy fetal development [10].

The dietitians at Taichung Veterans General Hospital, a Tertiary Medical Center have been providing nutrition counseling to pregnant women with diabetes for many years; however, the effectiveness of this counseling has not been thoroughly evaluated, nor have the common dietary issues among these women been systematically analyzed. Therefore, this study analyzes past electronic medical records from the hospital to investigate the effectiveness of nutritional therapy and to identify common dietary issues among pregnant women with diabetes. The findings aim to serve as a reference and guide for future nutrition education initiatives.

## Methods

### Study design and ethical approval

We conducted an observational retrospective analysis using electronic medical records of pregnant women aged 18 to 50 who were diagnosed with type 1 or type 2 diabetes before pregnancy from 2012 to 2022. The Institutional Review Board of Taichung Veterans General Hospital approved the protocol with a waiver for informed consent (IRB no. CE23103C, March 24, 2023). All research was conducted in accordance with the Declaration of Helsinki and the Declaration of Istanbul.

### Data collection and key variables

This study utilized de-identified electronic medical record data provided by the hospital’s information department. The data included patient age, type of diabetes (type 1 or type 2), mode of delivery (vaginal or cesarean), anthropometric measurements (height and gestational weight records), blood glucose control indicators (fasting blood glucose and glycated hemoglobin), and neonatal outcomes (birth weight, incidence of preterm birth, and macrosomia). Preterm birth was defined as delivery before 37 weeks of gestation, and macrosomia was defined as a birth weight greater than 4,000 g. In addition, information was collected on whether the patient received nutrition counseling during pregnancy, including the timing and frequency of the intervention, as well as changes in dietary intake before and after the counseling. The primary outcome of this study was the impact of nutrition counseling on dietary improvements among pregnant women with PDM. Secondary outcomes included whether these dietary changes contributed to improved maternal glycemic control and neonatal outcomes, such as the incidence of preterm birth and macrosomia.

Nutrition counseling was provided by registered dietitians at the hospital’s Department of Dietetics. For patients with known diabetes, counseling was typically conducted at the first prenatal visit. However, some patients were referred to our hospital for prenatal care during the mid to late stages of pregnancy; thus, counseling was conducted during the second or third trimester. Some patients, due to prior knowledge of dietary management during pregnancy, limited clinic visits, or scheduling conflicts, chose to decline referral for nutrition counseling. During the initial session, dietitians collected dietary information through structured interviews, including food portions and calculated macronutrient intake, which served as a representation of dietary intake before nutrition counseling (Before NC). In addition to individualized dietary assessment, the counseling included education on carbohydrate counting, selection of low glycemic index foods, meal planning based on the Taiwanese dietary guidelines and as well as recognition and management of hypoglycemic symptoms. Each session lasted approximately 30 min and was fully covered by Taiwan’s National Health Insurance, incurring no out-of-pocket cost for the patient. Dietitians followed up within two weeks to one month after the initial education session to assess dietary compliance and changes in dietary intake, which were used to represent post-counseling intake (After NC).

The number of nutrition counseling sessions varied depending on clinical judgment, patient availability, and physician referrals. Some patients received only one session due to late referral to our hospital for prenatal care, limited clinic visits, scheduling conflicts, or stable metabolic control. Others were scheduled for follow-up counseling sessions to reinforce dietary compliance or address glycemic fluctuations. Among the 24 participants with complete blood glucose records, 7 received nutrition counseling only once, while the remaining 17 received two or more sessions.

For biochemical data, blood glucose indicators during pregnancy were recorded, including fasting blood glucose and glycated hemoglobin (HbA1c). Blood samples were collected during routine prenatal visits according to physician instructions.

### Statistical analyses

We used Student’s t-test for continuous variables and the chi-squared test for categorical variables to determine differences between pregnant women who received nutrition counseling (NC group) and those who did not (Non-NC group). Paired t-tests were used to compare differences in dietary and nutrients intake, and blood glucose measurements before and after nutrition counseling. Subgroup analysis was conducted among participants with available pre- and post-intervention blood glucose data to evaluate the effect of counseling frequency. Paired t-tests were used within the one-time and repeated counseling groups to assess changes in fasting glucose and HbA1c, and the magnitude of change was compared between groups. A p-value < 0.05 was considered statistically significant.

## Results

### Participant characteristics

**Fig. 1:**
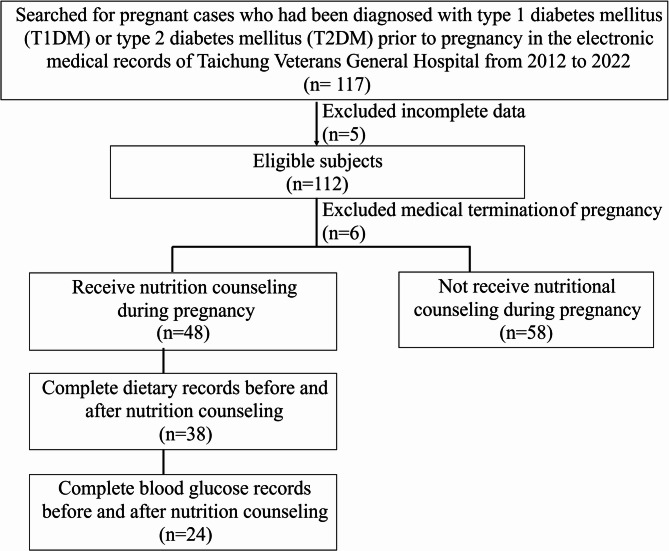
Flowchart of cases selection

We reviewed electronic medical records from Taichung Veterans General Hospital between 2012 and 2022, during which 11,468 women gave birth at the hospital. Among them, 117 were diagnosed with type 1 or type 2 diabetes prior to pregnancy, yielding a prevalence of approximately 1%. After excluding 5 cases with incomplete or irregular prenatal check-up records, 112 subjects were eligible. Among them, 6 cases experienced early termination of pregnancy due to medical reasons, leaving a total of 106 subjects included in the study. Of these, 48 received nutrition counseling, while 58 did not receive any nutrition counseling. Among the individuals who received nutrition counseling, 38 had complete dietary records before and after the intervention, while only 24 had complete blood glucose records before and after nutrition counseling. (Fig. 1).

The characteristics of the participants are listed in Table 1. The data indicated that there were no statistically significant differences in age, DM type, or mode of delivery between subjects who received nutrition counseling and those who did not. The proportion of cesarean deliveries was 66.7% in the with NC group and 69.0% in the Non-NC group. The pre-pregnancy BMI in the Non-NC group was significantly higher than that in the NC group (*p* = 0.020), indicating a potential baseline difference between groups. However, there was no statistically significant difference in weight gain during pregnancy between the two groups. In the with NC group, 62.5% of the participants received their first dietary education during the first trimester of pregnancy, and 14.6% received it during the third trimester.

**Table 1:** Basic information on pregnant women with pregestational diabetes mellitus (PDM)

	NC (*n* = 48)	Non-NC (*n* = 58)	*P* value
Age (year)	34.5 ± 4.6	34.3 ± 5.9	0.905
DM type			
Type 1 (%)	17 (35.4)	11 (19.0)	0.056
Type 2 (%)	31 (64.6)	47 (81.0)	
Mode of delivery (%)			
Vaginal Delivery	16 (33.3)	18 (31.0)	0.407
Cesarean Section	32 (66.7)	40 (69.0)	
BMI before pregnancy (kg/m^2^)	27.9 ± 8.0	32.4 ± 8.5	0.020*
Gestational weight gain	12.4 ± 6.5	9.4 ± 5.2	0.157
Timing of the first nutrition counseling			
First trimester	30 (62.5)		-
Second trimester	11 (22.9)		-
Third trimester	7 (14.6)		-

Data were presented as n (%) or mean ± SD. Differences between groups were tested using chi-square tests or student’s t test

*NC* nutrition counseling, *Non-NC* without nutrition counseling

*P* value < 0.05 was considered statistically significant

### Neonatal basic characteristics

The neonatal basic information is presented in Table 2. The proportion of full-term births was higher in the NC group (79.2%) than in the non-NC group (65.5%), although the difference was not statistically significant. (*p* = 0.120). Among full-term infants, there were no statistically significant differences between the two groups in gestational age, birth weight, or the proportion of fetal macrosomia. For preterm births, 10 cases (20.8%) occurred in the NC group and 20 cases (34.5%) in the non-NC group. Among preterm deliveries, the mean gestational age and birth weight were similar between groups. No cases of fetal macrosomia were observed in the NC group, whereas one case (5.0%) occurred in the non-NC group (*p* = 0.645). Regarding the onset of labour in preterm births, the proportions of spontaneous preterm birth, induction, and caesarean section did not significantly differ between the NC and non-NC groups. Among them, three cases in the NC group and five cases in the non-NC group experienced spontaneous preterm birth and ultimately delivered via caesarean section.

**Table 2:** Neonatal basic characteristics

	NC (*n* = 48)	Non-NC (*n* = 58)	*P* value
Full term (%)	38 (79.2)	38 (65.5)	
Weeks of gestation (week)	38.2 ± 1.0	38.4 ± 1.1	0.286
Birth weight (kg)	3.5 ± 0.4	3.3 ± 0.6	0.105
Fetal macrosomia (%)	7 (14.6)	6 (15.8)	0.500
Preterm (%)	10 (20.8)	20 (34.5)	0.120
Weeks of gestation (week)	32.6 ± 2.4	33.2 ± 3.3	0.642
Birth weight (kg)	2.1 ± 0.8	2.3 ± 1.1	0.601
Fetal macrosomia (%)	0 (0.0)	1 (5.0)	0.645
Spontaneous preterm births	5 (50.0)	9 (45.0)	0.550
Inductions	0 (0.0)	1 (5.0)	0.667
Caesarean sections	8 (80.0)	15 (75.0)	0.571

Data were presented as n (%) or mean ± SD. Differences between groups were tested using chi-square tests or student’s t test

*NC* nutrition counseling, *Non-NC* without nutrition counseling

*P* value < 0.05 was considered statistically significant

### Changes in dietary servings before and after nutrition counselings

Among the 48 cases who received nutrition counseling, 38 returned for follow-up and provided post-education dietary records. Following nutrition counseling, a significant increase in fruit and whole grain intake was observed (*p* = 0.014 and 0.009, respectively). Vegetable intake also showed a slight increase (*p* = 0.070), The results indicated a significant increase in the intake of fruits and whole grains, with servings increasing by 42% and 200%, respectively. Vegetable intake also showed a slight increase, while the consumption of sugar-sweetened beverages exhibited a decreasing trend (Table 3).

**Table 3:** Changes in dietary servings before and after nutrition counseling in complete dietary records on PDM

	Before NC (*n* = 38)	After NC (*n* = 38)	*P* value
Dairy products	1.1 ± 0.9	0.9 ± 0.9	0.241
Fruits	1.2 ± 1.1	1.7 ± 1.1	0.014 *
Vegetables	2.6 ± 1.1	3.0 ± 1.1	0.070
Total cereals	9.6 ± 3.7	9.6 ± 3.3	0.986
Whole grains	0.6 ± 1.3	1.8 ± 2.4	0.009 *
Lean meat	1.0 ± 1.3	1.4 ± 1.3	0.123
Medium-fat meat	5.6 ± 2.0	5.4 ± 1.7	0.661
High-fat meat	0.7 ± 1.1	0.6 ± 1.1	0.830
Oil, nuts and seeds	8.2 ± 3.0	8.3 ± 2.6	0.895
Sweeten beverage	0.6 ± 1.1	0.3 ± 0.7	0.291

Data were presented as mean ± SD

*NC* nutrition counseling

*Differences between before and after nutrition counseling (NC) were tested using paired t-test

*P* < 0.05 considered statistically significant

### Changes in dietary intake before and after nutrition counseling

Table 4 presents the changes in dietary intake before and after nutrition counseling during pregnancy. The results showed no significant differences in total caloric intake, protein, carbohydrate, and fat intake before and after nutrition counseling. The proportion of calories from saturated fat showed a decreasing trend, although it did not reach statistical significance. Notably, fiber intake significantly increased after nutrition counseling, indicating that the nutrition counseling effectively improved dietary fiber intake during pregnancy. The American Diabetes Association recommends that carbohydrate intake during pregnancy should not be less than 175 g per day. Our results showed that as many as 55.3% of pregnant women had carbohydrate intake below 175 g before receiving nutrition counseling. After the nutrition counseling intervention, the proportion of pregnant women with carbohydrate intake below 175 g decreased to 34.2%.

**Table 4:** Changes in dietary intake before and after nutrition counseling during pregnancy ^a^

	Before NC (*n* = 38)	After NC (*n* = 38)	*P* value
Calories (Kcal)	1714 ± 432	1743 ± 307	0.728
Protein (g)	71.7 ± 18.6	70.9 ± 14.5	0.828
Carbohydrate (g)	191.4 ± 58.0	195.6 ± 53.9	0.742
Carbohydrate < 175 g (%)	21 (55.3)	13 (34.2)	0.899
Fat (g)	73.5 ± 23.0	75.2 ± 15.9	0.675
SFA of total calories (%)	6.2 ± 2.7	5.4 ± 2.6	0.079
Fiber (g)	10.3 ± 4.6	14.3 ± 5.6	< 0.001*

Data were presented as mean ± SD

*NC* nutrition counseling, *SFA* saturated fatty acids

^*^Differences between before and after nutrition counseling (NC) were tested using paired t-test

*P* < 0.05 considered statistically significant

### The impact of dietary education during pregnancy on blood glucose changes

Since only 24 participants had blood glucose measurement data before and after the dietary education, Table 5 presents the blood glucose changes for these participants. The results showed significant improvements in fasting blood glucose and HbA1c levels after dietary education, with reductions of 18% and 11%, respectively. Of these 24 participants, 21 (87.5%) showed improvement in fasting plasma glucose, and 17 (70.8%) showed improvement in HbA1c levels. Among the participants, 14 had their insulin dosage adjusted by their physician at the time of referral to the dietitian. Therefore, the participants were further divided into those with insulin adjustments and those without. The results showed that participants with adjusted insulin dosages experienced significant improvements in fasting plasma glucose levels and HbA1c levels after dietary education, with reductions of 15.5% and 10.5%, respectively. Participants without insulin adjustments also showed a significant improvement in fasting plasma glucose levels, with a reduction of 21.8%.

**Table 5:** Changes in blood glucose levels before and after dietary education ^a^

	Before NC	After NC	*P* value
Total subjects (*n* = 24)			
Fasting plasma glucose (mg/dL)	129.5 ± 35.5	106.1 ± 32.2	< 0.001*
HbA1c (%)	7.3 ± 1.3	6.5 ± 0.8	0.001*
With insulin dose modification (*n* = 14)			
Fasting plasma glucose (mg/dL)	133.3 ± 33.8	112.7 ± 34.7	0.018*
HbA1c (%)	7.6 ± 1.4	6.8 ± 0.9	0.013*
Without insulin dose modification (*n* = 10)			
Fasting plasma glucose (mg/dL)	124.1 ± 39.0	97.0 ± 27.3	0.005*
HbA1c (%)	6.7 ± 1.0	6.1 ± 0.5	0.064

^a^Data were presented as mean ± SD

^*^Differences between before and after nutrition counseling (NC) were tested using paired t-test

*P* < 0.05 considered statistically significant

### The impact of the frequency of nutrition counseling on blood sugar changes during pregnancy

Among the 24 cases with blood glucose records, 7 received nutrition counseling only once, 17 received it more than once. The impact of the frequency of nutrition counseling on blood glucose improvement is presented in Table 6. Although different frequencies of nutrition counseling improved fasting plasma glucose and HbA1c levels, only cases receiving nutrition counseling more than once showed significant improvements, with reductions of 19.3% in fasting plasma glucose and 10.0% in HbA1c. This suggests that multiple sessions of nutrition counseling help individuals better understand dietary control concepts and effectively implement dietary control.

**Table 6:** Effect of the frequency of nutrition counseling on blood sugar changes during pregnancy

Covariate	Before NC	After NC	*P* value
One time (*n* = 7)			
Fasting plasma glucose (mg/dL)	118.5 ± 37.0	101.1 ± 26.3	0.101
HbA1c (%)	7.4 ± 1.5	6.8 ± 1.1	0.094
More than once (*n* = 17)			
Fasting plasma glucose (mg/dL)	134.0 ± 35.0	108.2 ± 34.9	0.001*
HbA1c (%)	7.2 ± 1.2	6.5 ± 0.7	0.008*

^a^Data were presented as mean ± SD

^*^Differences between before and after nutrition counseling (NC) were tested using paired t-test

*P* < 0.05 considered statistically significant

## Discussion

This study found that nutrition counseling had no significant impact on obstetric and neonatal outcomes, including gestational weight gain, preterm birth, and macrosomia. However, it was associated with improved maternal glycemic control, particularly among those who received multiple counseling sessions. In terms of dietary improvement, the intake of fruits, whole grains, and dietary fiber increased significantly (*p* < 0.001), while the proportion of women consuming less than 175 g of carbohydrates showed a decreasing trend. Among participants with blood glucose records, only those who received repeated counseling demonstrated significant metabolic improvements, with fasting glucose reduced by 19.3% and HbA1c by 10.0%. These findings suggest that the frequency of counseling may play a critical role in helping pregnant women with PDM better understand and implement dietary strategies for glycemic control.

Previous studies have shown that pregnant women with PDM face a higher risk of perinatal complications compared to those without diabetes. However, dietary management can improve their blood glucose control and reduce the risks of preterm birth and macrosomia. Alexopoulos et al. highlighted that PDM, a chronic metabolic disorder, carries greater pregnancy risks than gestational diabetes, including preterm birth, macrosomia, stillbirth, and congenital anomalies. Therefore, strict preconception metabolic assessment and glycemic control (target HbA1c < 6.5%) are essential. During pregnancy, individualized medical nutrition therapy (MNT), insulin management, and continuous monitoring of diet, weight, and glucose are recommended. Preconception care significantly reduces fetal structural abnormalities and improves perinatal outcomes, underscoring the importance of multidisciplinary involvement throughout pregnancy [11].

The prevalence of diabetes mellitus in pregnancy (DIP) is 15.8% among all pregnant women. Among these cases, the majority are gestational diabetes mellitus (GDM), accounting for 91.8%, followed by preexisting diabetes mellitus (PDM) at 5.7%, and a smaller portion are newly diagnosed diabetes during pregnancy, comprising 2.5% [12]. We aim to evaluate the effectiveness of nutrition counseling on blood glucose control and related outcomes in women with PDM. Due to the limited number of cases, conducting a prospective evaluation would require a long data collection period. Therefore, this study adopts a retrospective chart review approach to assess the impact of past nutrition counseling interventions.

According to the 2023 Birth Notification Annual Report by the Ministry of Health and Welfare, the average preterm birth rate among live newborns in Taiwan over the past decade is approximately 10% [13]. In this study, the preterm birth rate among women with PDM was 21% for those who received nutrition counseling and 35% for those who did not—both significantly higher than the rate among the general pregnant population. These findings indicate that women with PDM have a higher risk of preterm birth and should be considered a high-risk group requiring close medical attention.

In Taiwan, pregnant women are covered by National Health Insurance for up to 12 prenatal checkups, allowing for perinatal monitoring every 2 to 4 weeks on average at medical institutions. However, nutrition counseling is not a routine component of prenatal care. It is typically provided only when the patient is diagnosed with gestational diabetes with poor glycemic control, hypertension, or other high-risk pregnancy conditions, in which case they are referred to a dietitian. In our hospital, nutrition counseling was available to all pregnant women with PDM through physician referral. However, patients had the autonomy to decide whether to participate. Taiwan’s healthcare system also supports multidisciplinary diabetes care through the national Diabetes Shared Care Network (P4P), which includes reimbursement for nutrition counseling. Our findings revealed that women who did not receive formal nutrition counseling presented with a higher baseline BMI. Prior evidence supports the adverse impact of maternal adiposity on pregnancy outcomes. Kong et al. reported that moderately obese women with type 2 diabetes had an elevated risk of delivering Large for gestational age (LGA) infants and preterm birth, while Siegel et al. demonstrated that gestational weight gain exceeding the IOM recommendations was significantly associated with increased risks of LGA and macrosomia [14, 15]. In contrast, although our non-counseled group exhibited a higher baseline BMI, their gestational weight gain was comparatively lower, and the rates of preterm birth and macrosomia did not increase significantly. One possible explanation is that these women may have received informal dietary advice from other sources, such as outpatient healthcare providers, even though no standardized dietary records were available. The absence of detailed dietary counseling data limits our ability to further evaluate this possibility.

Over the 10 years of medical record review, we did observe a gradual increase in the number of PDM cases, particularly type 2 diabetes mellitus (T2DM). We believe this may be related to the trend of earlier onset of diabetes and delayed childbearing age. The number of referrals for nutrition counseling also increased over time, in parallel with the rising number of PDM cases. The overall proportion of patients receiving nutrition counseling increased from an average of 35% in the first five years to 51% in the latter five years, indicating a growing trend in physician referrals for dietitian services over the 10-year period.

Among the 48 PDM pregnant women who received dietary education, only 38 returned for follow-up dietary assessments. The results showed a significant increase in the consumption of fruits and whole grains, along with a trend toward higher vegetable intake. These changes were reflected in a notable improvement in dietary fiber intake. Studies have shown that dietary fiber obtained from whole grains, vegetables, and fruits can help with blood glucose control [16]. The American Diabetes Association (ADA) recommends that pregnant women with diabetes consume 28 g of dietary fiber per day [17], and Taiwan’s guidelines offer similar recommendations. However, our findings indicated that most pregnant women did not meet the recommended intake levels. Although there was a significant improvement following dietary education, the intake still fell short of the recommended amount. This highlights the need for a stronger focus on dietary fiber intake in future nutrition counseling for women with PDM.

Lin et al. reported that the average carbohydrate intake among general pregnant women in Taiwan (without pregestational diabetes) is approximately 330 g per day, accounting for about 60% of total energy intake [18]. In our study, the average carbohydrate intake among women with PDM was 191 g per day, accounting for 45% of total energy intake. The Taiwan Clinical Guidelines for Gestational Diabetes Care, published in 2020, recommend that carbohydrate intake should make up 40% to 50% of total energy, with an emphasis on choosing low-glycemic index (GI) foods and avoiding refined sugars such as sucrose. Therefore, although the proportion of carbohydrate intake in PDM mothers is lower than that of the general pregnant population, it still falls within the recommended range of the national guidelines.

The American Diabetes Association (ADA) recommends that pregnant women with diabetes consume no less than 175 g of carbohydrates per day. This intake ensures an adequate supply of glucose to meet the basic energy needs of both the mother and the developing fetus, and helps prevent ketone production, which is important for supporting normal fetal neural development [17]. However, our results showed that before receiving nutrition counseling, more than half of the pregnant women with PDM consumed less than 175 g of carbohydrates per day, likely due to concerns about elevated blood glucose levels. Interestingly, none of the PDM pregnant women with inadequate carbohydrate intake were found to have ketonuria during their prenatal checkups. Mijatovic et al. found that a daily intake of approximately 165 g of carbohydrates did not raise ketone to a clinically significant levels in patients with gestational diabetes mellitus (GDM) [19]. We speculate that the absence of ketonuria during prenatal checkups in most participants of this study may be since the majority did not consume an extremely low-carbohydrate diet. Nevertheless, low-carbohydrate diets during pregnancy may lead to inadequate intake of essential micronutrients, including folate, iron, calcium, vitamin D, iodine, and selenium [20]. Therefore, it is strongly recommended that women with PDM consume appropriate amounts of carbohydrates from sources such as whole grains, vegetables, fruits, and milk.

Blood glucose testing is typically ordered at the discretion of the attending physician; therefore, not all individuals who received nutrition counseling had both pre- and post-education blood glucose measurements. Among the 24 participants with available data before and after nutrition counseling significant improvements were observed in both blood glucose levels and HbA1c. We further noted that, in some cases, physicians adjusted insulin dosages at the time of referral for nutrition counseling. Since insulin adjustment can also influence blood glucose levels, we analyzed the data by separating cases with insulin dosage adjustments from those without. The results showed that, prior to receiving nutrition counseling, women whose insulin dosages were adjusted had higher fasting blood glucose and HbA1c levels compared to those without dosage changes. This finding supports the clinical decision to modify insulin therapy for these individuals and indicates that they subsequently experienced greater improvements in glycemic control. Interestingly, significant improvements in blood glucose levels were also observed among participants whose insulin dosages were not adjusted. Our findings are consistent with previous studies showing that selecting appropriate amounts of fiber-rich carbohydrates—such as whole grains, vegetables, and fruits—can help improve glycemic control in pregnant women with PDM [16, 21, 22].

A retrospective study found that GDM patients who received multiple nutrition counseling sessions experienced reductions in HbA1c levels, as well as decreased risks of neonatal macrosomia and pregnancy-related complications [23]. Another study evaluated the association between medical nutrition therapy (MNT) consultations and GWG in Mexican women with type 2 diabetes (T2DM) and gestational diabetes mellitus (GDM). The results showed that each additional MNT consultation was associated with a 1.2 kg reduction in GWG (β = −1.2; 95% CI: −2, −0.3; *p* = 0.007) [24]. Our results also showed that participants who received more than one nutrition counseling session experienced significantly greater improvements in glycemic control compared to those who received only one session. This trend was also reflected in maternal gestational weight gain (11.3 ± 6.0 kg vs. 15.9 ± 6.8 kg, *p* = 0.113), infant birth weight (3.5 ± 0.4 kg vs. 3.7 ± 0.5 kg, *p* = 0.149), and the incidence of macrosomia (14% vs. 30%, *p* = 0.271), although these differences did not reach statistical significance and should be regarded as exploratory findings rather than definitive evidence.

This study is the first in Taiwan to examine the effects of nutrition counseling on blood glucose control and neonatal outcomes among women with PDM. The results confirm that repeated nutrition counseling significantly improves glycemic parameters and enhances dietary quality, especially in terms of fiber intake. Although improvements in gestational weight gain and neonatal outcomes were not statistically significant, they were clinically meaningful and favored participants who received multiple nutrition sessions. Our study has several limitations that warrant cautious interpretation. The retrospective design and modest sample size may restrict the generalizability of our findings, and no prior power analysis was performed. Nevertheless, based on the observed changes in fasting glucose levels, a post hoc calculation indicated that a sample of at least 20 participants would achieve 80% power at α = 0.05, suggesting that the primary analysis was adequately powered, although some subgroup comparisons may have been underpowered. Despite the relatively small cohort, the study included all eligible cases of pregestational diabetes mellitus (PDM) over a 10-year period, representing approximately 1% of all pregnancies—consistent with the national prevalence of PDM in Taiwan (≈ 0.9%) [23]. Baseline differences, particularly the higher pre-pregnancy BMI in the Non-NC group, may have introduced confounding and limited the extent to which observed outcomes can be attributed solely to nutrition counseling. Incomplete dietary and biochemical records further reduced statistical power and external validity. The absence of longitudinal biochemical data in the Non-NC group also restricted direct comparisons of metabolic changes. These factors, coupled with the retrospective nature of the study, may have led to selection bias. Despite these limitations, the retrospective design provided a practical framework to preliminarily examine the effects of nutrition counseling in this rare population, where prospective data collection is time-intensive. Future multicenter, prospective studies with standardized dietary and biochemical assessments are needed to confirm the benefits of nutritional interventions on maternal and neonatal outcomes. Moreover, electronic medical record–based analyses cannot fully capture behavioral changes or dietary adherence, and the limited dietary data prevented detailed assessment of micronutrient adequacy. In conclusion, although constrained by design and data limitations, our findings underscore the potential importance of structured, repeated nutrition counseling in the management of PDM and provide a basis for future clinical programs and policy development.

## Conclusions

This retrospective study indicated that nutrition counseling can enhance fiber intake by increasing the consumption of fruits, vegetables, and whole grains. Repeated counseling sessions were significantly associated with reductions in fasting glucose and glycated hemoglobin (HbA1c) levels. Although changes in gestational weight gain, infant birth weight, and the incidence of macrosomia did not reach statistical significance, the findings underscore the value of structured and continuous nutritional support as an essential component of prenatal care for women with PDM.

## Data Availability

The data supporting the reported results and conclusions can be found in the submitted figure and tables. Additional research materials and protocols that are relevant to the study are available from the corresponding author upon reasonable request.
